# Gold Nanoclusters for Bacterial Detection and Infection Therapy

**DOI:** 10.3389/fchem.2020.00181

**Published:** 2020-03-24

**Authors:** Mingxiu Tang, Jian Zhang, Chunyan Yang, Youkun Zheng, Hui Jiang

**Affiliations:** ^1^The Second Affiliated Hospital, Affiliated Traditional Chinese Medicine Hospital, Southwest Medical University, Luzhou, China; ^2^Key Laboratory of Medical Electrophysiology of Ministry of Education, Drug Discovery Research Center, Southwest Medical University, Luzhou, China; ^3^State Key Laboratory of Bioelectronics, School of Biological Science and Medical Engineering, Southeast University, Nanjing, China

**Keywords:** gold nanoclusters, photoluminescence, bacteria detection, antibacterial activity, multidrug-resistant bacteria

## Abstract

Infections caused by antibiotic-resistant bacteria have become one of the most serious global public health crises. Early detection and effective treatment can effectively prevent deterioration and further spreading of the bacterial infections. Therefore, there is an urgent need for time-saving diagnosis as well as therapeutically potent therapy approaches. Development of nanomedicine has provided more choices for detection and therapy of bacterial infections. Ultrasmall gold nanoclusters (Au NCs) are emerging as potential antibacterial agents and have drawn intense attention in the biomedical fields owing to their excellent biocompatibility and unusual physicochemical properties. Recent significant efforts have shown that these versatile Au NCs also have great application potential in the selective detection of bacteria and infection treatment. In this review, we will provide an overview of research progress on the development of versatile Au NCs for bacterial detection and infection treatment, and the mechanisms of action of designed diagnostic and therapeutic agents will be highlighted. Based on these cases, we have briefly discussed the current issues and perspective of Au NCs for bacterial detection and infection treatment applications.

## Introduction

The prevalence of pathogenic bacteria, especially multidrug-resistant bacteria, has become a serious global health crisis (Blair et al., 2015). Conventional antibiotics often appear to be incapable of responding to the prevalence of multidrug-resistant bacteria, either ineffective or inducing the emergence of new resistance after a period of use (Huh and Kwon, 2011). In particular, the emergence of ESKAPE superbugs even worsens the situation (Boucher et al., 2009). According to a statement from the Centers for Disease Control and Prevention, the world is on the verge of entering the “post-antibiotic era,” one where the death toll from bacterial infections than from cancer (Gupta et al., 2019). Therefore, there is an urgent need to develop alternative therapeutically effective antibacterial agents that are powerful and cost-effective enough to fight multidrug-resistant bacterial infections.

In addition to the treatment, the effective diagnosis of multidrug-resistant bacterial infections is also a huge challenge. Accurate and early detection of pathogenic bacteria is critical to identify infectious disease. Current techniques to detect bacteria include culture-dependent method, biochemical assays, PCR and sequencing, which are expensive and time-consuming (Lazcka et al., 2007; Ray et al., 2012; Yuan et al., 2018; Li D. et al., 2019). The lack of timely diagnosis has further worsened the condition of many patients with bacterial infections (Palestro and Love, 2009).

**Scheme 1:**
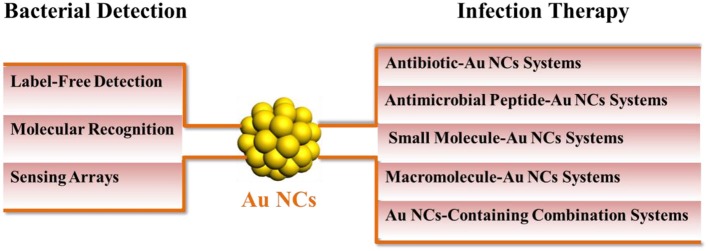
An overview of Au NCs-based bacterial infection diagnostic and therapeutic strategies.

To overcome the drawbacks of conventional infection diagnostic and therapeutic strategies, various engineered nanomaterials have been used for diagnosis and treatment of bacterial infections (Disney et al., 2004; Kulagina et al., 2005; Yuan et al., 2014, 2018; Mahlapuu et al., 2016). Among these alternative agents, ultrasmall metal nanoclusters, in particular Au NCs, have attracted significant attention for diagnosis and treatment of bacterial infections. Gold-based NCs have intrinsic advantages such as facile syntheses, extremely large surface area, excellent biocompatibility, strong photoluminescence, high photostability, and easy functionalization with other biomolecules. Benefits from these excellent physicochemical properties, Au NCs have great promise in biomedical applications, such as sensing, imaging, and diseases treatment (Chen L. Y. et al., 2015; Zheng Y. et al., 2017; Hu et al., 2018; Chen et al., 2019). The antibacterial activity of Au NCs has been also innovatively explored over the past few years (Zheng K. et al., 2017; Zheng et al., 2018a,c; Xie et al., 2018). Apart from antibacterial activity, unusual photoluminescence properties of Au NCs also provide potential applications for their use as detection/imaging agents for bacterial pathogens (Chan and Chen, 2012; Zheng et al., 2018d; Li D. et al., 2019). Obviously, both diagnosis and treatment are essential to control the prevalence of multidrug-resistant bacterial infections. Moreover, the in-depth understanding of the fundamental principles of diagnosis and treatment plays a key role in designing bacterial biosensors and antimicrobial agents. In this review, we will summarize the efforts of Au NCs for diagnosis and treatment of bacterial infections in the recent decade as Au NCs may provide solutions to address these intractable challenges for bacterial infections (Scheme 1). Based on the overview of Au NCs, we firstly summarize the recent progress of Au NCs for bacterial detection, containing the probes design, sensitivity, and selectivity of miscellaneous gold-based NCs. Then we discuss the antibacterial activity on basis of the mechanisms by different Au NCs. Physicochemical properties of Au NCs such as surface chemistry, photoluminescence, and size that affect the antibacterial behavior or detection performance are analyzed to offer insight on the further rational design of new diagnostic and therapeutic agents. Finally, a brief discussion of current problems and future developments of Au NCs for diagnosis and treatment of bacterial infections is provided.

## ABOUT Au NCs

Au NCs refer to gold species containing a few to several hundred Au atoms, with their dimensions below a critical size for electronic energy quantization. According to the free-electron model, the critical size for Au is ~2 nm, which is comparable to the Fermi wavelength of electrons (Zhang and Wang, 2014; Yang et al., 2015). In this size regime, the strong quantum confinement of free electrons leads to the discrete electronic states and thus Au NCs exhibit molecule-like properties, such as HOMO-LUMO transition, large Stokes shift, and strong photoluminescence (Goswami et al., 2016; Song et al., 2016). Au NCs show dramatically different optical and chemical properties from those of larger gold nanoparticles (NPs). For example, Au NCs do not possess surface plasmon resonance (SPR) absorption in the visible region but exhibit apparent fluorescence emission in the near-infrared (NIR) to visible region (Zheng Y. et al., 2017). In addition to ultrasmall size, many studies have also revealed that the optical properties of Au NCs highly depend on their structures, oxidation states, and surface ligands as well as environmental parameters such as temperature, pH, and ionic strength (Zheng Y. et al., 2017; Chen et al., 2019). As a bridge between single Au atom and plasmonic NPs, Au NCs have received increasing attention in many fields, including bacterial detection described in the following sections. Up to now, several reviews have been dedicated to the ultrasmall Au NCs (Luo et al., 2014; Jin et al., 2016; Zheng Y. et al., 2017; Chen et al., 2019).

To prepare stable and high quality Au NCs, z, polymers, peptides, DNA, and proteins that act as capping agents are required when using various synthetic methods, including chemical reduction, photoreduction, electroreduction, and chemical etching (Zheng Y. et al., 2017). To further benefit and broaden applications of Au NCs, it is necessary for further functionalization with surface ligands (e.g., folic acid, proteins) on Au NCs, commonly via ligand exchange, bioconjugation, and non-covalent interaction (Jin et al., 2016; Song et al., 2016; Zheng Y. et al., 2017).

In addition to the advantages of easy preparation and unique physicochemical properties, the excellent biocompatibility is also the reason why Au NCs have received widespread attention, especially in biomedical fields. Indeed, as the “noblest” metals, gold, is inert, highly stable, and would not easily dissociate into ions (Hammer and Norskov, 1995). These features contribute to the widely accepted notion of Au NPs as being highly biocompatible in mammalian system, both *in vitro* and *in vivo* (Connor et al., 2005; Lewinski et al., 2008). This biocompatibility in mammalian cells still remains when the size locates in the range of NCs (Pan et al., 2007; Li et al., 2016). For example, utilizing the *in vitro* multiple cell models, no cytotoxic effect was observed on the cells exposed with Au NCs (Zheng et al., 2018b). Conversely, they were found to improve cell metabolism and overall cell proliferation. In animal studies, they show improved tumor uptake and high renal clearance (Zhang et al., 2012; Liang et al., 2017; Yu et al., 2019). Interestingly, they showed significant cytotoxicity against prokaryotic bacterial cells as opposed to eukaryotic mammalian cells. This selective cytotoxicity may be resulted from the limitations of lysosomal phagocytosis and mitochondrial obstacles in mammalian cells (Marrache and Dhar, 2012). Note that it is always critical to tightly control the surface properties of Au NCs as they can potentially affect the toxicity.

## Bacterial Detection With Gold-Based Ncs

### Label-Free Detection of Bacteria

**Figure 1:**
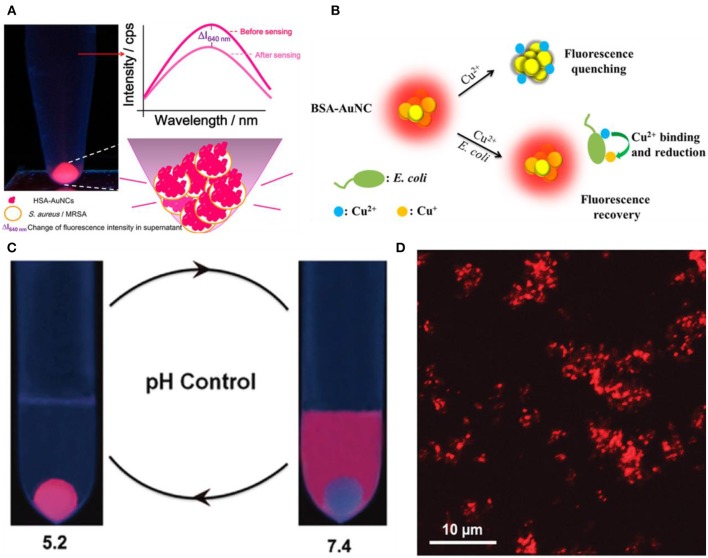
**(A)** Luminescent HSA-Au NCs as selective probes for *Staphylococcus aureus* and MRSA. Reproduced from Chan and Chen (2012) with permission from American Chemical Society. **(B)** Schematic illustration of the working principle for the Cu^2+^ mediated on-off-on Au NC-based fluorescent probe for rapid *Escherichia coli* detection. Reproduced from Yan et al. (2018) with permission from American Chemical Society. **(C)** Simplified scheme of pH controllable adherence of CP-GNC to *E. coli* cells. Specially, CP-GNC was fully attached to the cells at pH 5.2, whereas all the CP-GNC detached from the surface of *E. coli* cells at pH 7.4. **(D)** Bacterial cells can be efficiently labeled and form cell clusters using CP-GNC. Reproduced from Liu P. et al. (2015) with permission from Wiley-VCH Verlag & Co. KGaA, Weinheim.

Depending on the specific fluorescence changes caused by bacterial cells, photoluminescent gold-based NCs can be employed for label-free fluorescence detection of bacteria. For example, Chan and Chen found that human serum albumin protected gold nanoclusters (HSA-Au NCs) can act as selective fluorescent probes for *S. aureus* and methicillin-resistant *S. aureus* (MRSA) (Chan and Chen, 2012). HSA-Au NCs can bind to *S. aureus* and MRSA with high specificity, resulting in a significant fluorescence enhancement (Figure 1A). In another study, Yan and coworkers designed an on-off-on probe based fluorescent Au NC for rapid and selective detection of *Escherichia coli*, by hijacking the unique Cu^2+^-binding and redox pathways of *E. coil* to recover the photoluminescence of Au NC from copper-caused quenching (Figure 1B) (Yan et al., 2018). Based on this fluorescent probe, it can successfully allow the rapid determination and detection of *E. coli* in artificially contaminated water with trace concentrations of bacteria (89 CFU/mL) within 30 min, showing great application prospects for rapid point-of-care analysis of pathogenic *E. coli* in environment monitoring and clinical diagnosis.

**Figure 2:**
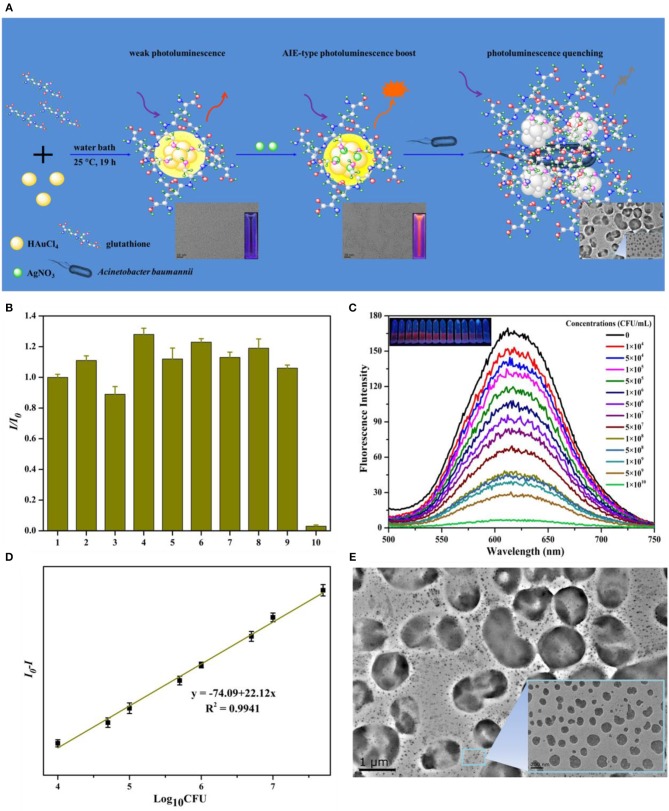
**(A)** Schematic illustration of the syntheses of AuAg NCs and photoluminescent quenching by *Acinetobacter baumannii*. **(B)** The fluorescence of AuAg NCs was selectively quenched by *A. baumannii*. The order numbers from 1 to 10 in turn represent the group in the presence of no bacteria (control), *Bacillus mycoides, Staphylococcus aureus*, methicillin-resistant *S. aureus, Candida albicans, P. aeruginosa, E. coli*, vancomycin-resistant *Enterococcus faecium, Saccharomyces cerevisiae*, and *A. baumannii*, respectively. **(C)** The concentration dependent quenching effect of *A. baumannii* toward AuAg NCs. Insets: Digital photos of AuAg NCs under UV illumination after treatment with different concentrations *A. baumannii*. **(D)** Relative fluorescence intensity (*I*_0_*-I*) of AuAg NCs in contrast to the logarithm of the *A. baumannii* concentrations. **(E)** The transmission electron microscopy (TEM) micrographs of photoluminescent AuAg NCs treated with 1 × 10^5^ CFU/mL *A. baumannii* demonstrate the bacteria induced agglomeration of NCs. Reproduced from Zheng et al. (2018d) with permission from Elsevier Ltd.

On the other hand, pH-responsive Au NCs can control the labeling of bacterial cells through pH regulation. Liu et al. developed a new method for the preparation of cross-linked protein (bovine serum albumin, BSA) with Au NCs (CP-GNC) (Liu P. et al., 2015). BSA is a typical amphoteric electrolyte, which means the surface net charges of CP-GNC can be adjusted by environmental pH due to the gain or loss of protons. Since bacterial cells are mainly negatively charged, the adhesion to and release of CP-GNC from *E. coli* cells can be easily controlled via modulating the pH (Figure 1C). Notably, CP-GNC-based fluorescent probe provides a solution for the label-free detection of *E. coli*. The confocal microscopy images showed that bacteria were efficiently labeled by this probe and formed cell clusters at pH 5.2 (Figure 1D). In another work, the antimicrobial peptide stabilized Au NCs also exhibits pH-responsive bacterial binding effect, which were useful for the fluorescence detection and imaging of bacterial infection (Pranantyo et al., 2019). Our recent study showed that the photoluminescence intensity of thiolated Au NCs can be significantly enhanced by silver ion doping (Zheng et al., 2018d). The strong photoluminescence of AuAg NCs (Ag-doped Au NCs) can be selectively and rapidly quenched by *Acinetobacter baumannii* via agglomeration of NCs, which allows the label-free detection of *A. baumannii* with a limit of detection (LOD) of 2.3 × 10^3^ colony forming unit (CFU)/mL (Figure 2). This study may provide a rapidly alternative strategy for the analysis of *A. baumannii* in clinical samples. Nevertheless, further study is still essential to produce an updated version of these materials with high selectivity and sensitivity toward specific bacterial species by combining bacteria recognizing components.

### Recognition Through Molecular Motifs

**Figure 3:**
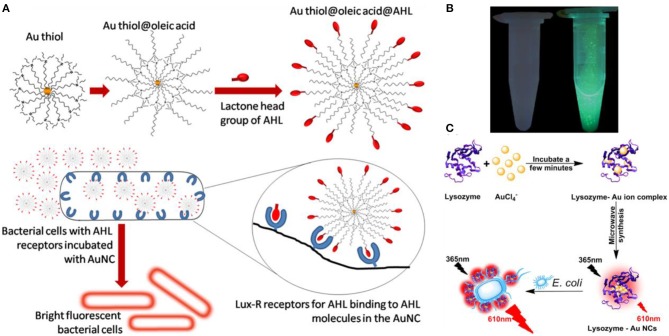
**(A)** Interaction of the fluorescent probe with bacterial cells: structure of the probe with AHL signal molecules deployed on the surface with lactone and amide moieties intact (top) and specific binding of AHL head groups to receptor sites in Lux-R regulators within bacteria (bottom). Reproduced from Mukherji et al. (2013) with permission from American Chemical Society. **(B)** Visualization of mannose-protected Au NCs (25 nM) in the absence (left) and presence (right) of *E. coli* (2.5 × 10^8^ CFU/mL) upon excitation under a hand-held UV lamp (365 nm). Reproduced from Tseng et al. (2011) with permission from Elsevier B.V. **(C)** Schematic diagram of the synthesis of the red fluorescent lysozyme-Au NCs and fluorescence enhancement detection of *E. coli*. Reproduced from Liu J. et al. (2015) with permission from Elsevier B.V.

The main drawback of using fluorescence Au NCs for bacterial detection is that their selectivity is generally non-ideal (Chen et al., 2019). To significantly improve the selectivity and efficiency, the common strategy is to decorate the clusters with ligands that recognize receptors on bacterial cells. For instance, Mukherji and coworkers functionalized Au NCs with acyl homoserine lactone (AHL) quorum sensing signal molecules that could recognize the Lux-R family regulators in *E. coli* (Figure 3A) (Mukherji et al., 2013). This decoration allows differentiation of *E. coli* from *S. aureus* suspensions that do not produce this special receptor. Khlebtsov et al. used highly fluorescent BSA-capped Au NCs decorated with human antistaphylococcal immunoglobulin (antiSAlgG) for targeted detection of *S. aureus* in bacterial mixtures (Khlebtsov et al., 2015). Compared with non-specific electrostatic binding of HSA-Au NCs to *S. aureus* at pH around 5–6, this biosensor can show an enhanced selectivity at the physiological pH of 7.4.

Type 1 fimbriae present on the surface of *Enterobacteriaceae*, such as *E. coil*, are responsible for their mannose- and mannoside-binding active sites (Soto and Hultgren, 1999; Harris et al., 2001). This family of proteins contains FimA, FimF, FimG, and FimH, and FimH is uniquely responsible for the binding to mannose. Mannose-etched Au NCs were used as special recognizer to develop a simple approach for fluorescence detection of *E. coli* (Huang et al., 2009; Tseng et al., 2011). The Au NCs bind to *E. coli* through the multivalent interactions between the NCs and FimH on the bacterial pili of *E. coli*, resulting in brightly fluorescent cell clusters (Figure 3B). The fluorescent signal was linearly proportional to the bacterial concentration, monitoring the fluorescence changes of Au NCs allowed the detection of *E. coli* with a LOD of 150 CFU/mL (Tseng et al., 2011). In addition, microwave-assistant synthesized mannose-protected Au NCs are also capable of selectively detecting the *E. coli* J96, a urinary tract infection isolate, by binding to FimH protein expressed on the type 1 pili (Chan et al., 2013). A similar recognition mechanism was also employed to establish specific probe for detection of *Listeria monocytogenes* (Hossein-Nejad-Ariani et al., 2018).

Besides surface receptors, enzymes such as lysozyme can recognize bacteria by binding to their specific site on cell surface (Li D. et al., 2019). Therefore, lysozyme-decorated Au NCs may be used for specific identification of bacteria. For instance, a point-of-care detection strategy for analysis bacteria has been established by using lysozyme-protected Au NCs that are prepared through a one-pot synthesis and reserved specific identification capability for *E. coli* (Liu J. et al., 2015). Based on the specific recognition, lysozyme-decorated Au NCs could selectively anchor onto the surface of *E. coli*, leading to strong red photoluminescence boost (Figure 3C). This strategy should be generalizable, and fluorescent Au NCs decorated with other recognition motifs could also be used to sense pathogenic bacteria.

**Figure 4:**
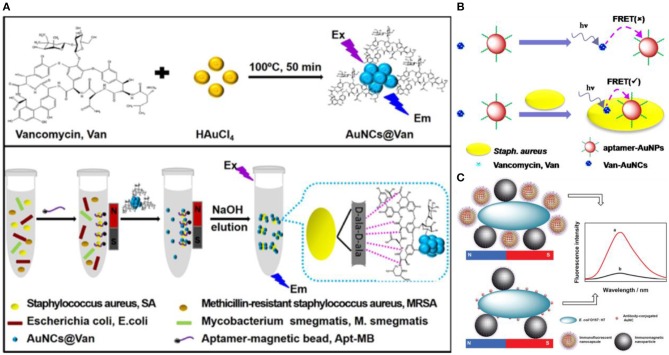
**(A)** Schematic illustrations of (top) one-step preparation of Au NCs@Van and (bottom) determination of *S. aureus* in mixtures using the aptamer-coated magnetic beads and Au NCs@Van dual recognition strategy. Reproduced from Cheng et al. (2016) with permission from American Chemical Society. **(B)** Illustration of the vancomycin and aptamer dual-recognition molecule based FRET assay platform for *S. aureus*. Reproduced from Yu et al. (2017) with permission from American Chemical Society. **(C)** Illustration of the immunoassay of *E. coli* O157:H7 using Au NCs@CS nanocapsules and Au NCs as labels. Reproduced from Cheng et al. (2018) with permission from The Royal Society of Chemistry.

To further improve the selectivity and sensitivity of bacterial detection, dual recognition probes based on fluorescent Au NCs have also been developed. Song and coworkers developed a dual recognition approach that integrates DNA aptamer and antibiotic-based dual recognition units, which enables sensitive and selective fluorescent detection of *S. aureus* in presence of ultrahigh concentrations of other bacteria strains (Figure 4A) (Cheng et al., 2016). Aptamer-decorated magnetic beads were used for specific capture of *S. aureus*. In another work, vancomycin-stabilized fluorescent Au NCs (Au NCs@Van) were employed for sensitive quantification of *S. aureus* with a LOD of 16 CFU/mL by measuring their photoluminescence intensity. Indeed, vancomycin can combine with *S. aureus* by binding onto terminal D-alanyl-D-alanine residues of N-acetylmuramic acid and N-acetylglucosamine peptide subunits on the cell wall of the gram-positive bacteria, which will make the Au NCs adhere to the surface of *S. aureus* (Xing et al., 2002; Chung et al., 2011). Using this strategy, about 70 CFU/mL of *S. aureus* in complex samples could be successfully sensed. Relying on vancomycin and aptamer as dual recognition molecules, Song et al. further generalized a universal strategy for selective detection of *S. aureus* using a dual-recognition motif-based fluorescence resonance energy transfer (FRET) platform (Figure 4B) (Yu et al., 2017). Within 30 min, by using Au NCs@Van and aptamer-modified Au NPs as the energy donor and acceptor, respectively, the FRET signal shows a linear variation with the concentration of *S. aureus* in the range from 20 to 108 CFU/mL with a LOD of 10 CFU/mL. This dual-recognition FRET strategy showed recoveries from 99.00% to the 109.75% for sensing *S. aureus* in real samples, which have great application potential in infectious disease diagnosis and environmental monitoring. In another study, nanocapsules with antibody-functionalized Au NCs combined in chitosan (Au NCs@CS) and immunomagnetic NPs were employed to ultrasensitive recognize *E. coli* O157:H7 (Figure 4C) (Cheng et al., 2018). After separation by magnetic fields, *E. coli* O157:H7 were isolated attached to the immunomagnetic NPs and quantified by the fluorescent changes of Au NCs@CS linked to bacteria.

In addition, a recent study showed that the mimic enzyme catalytic properties of Au NCs can also be exploited for colorimetric differentiation of pathogenic bacteria (Xie et al., 2019). A UV-assisted peroxidase-like Au NC sensor with an aptamer specific to *S. aureus* was developed. *S. aureus* was attached to the probe, which allows the catalyzed decomposition of hydrogen peroxide to hydroxyl radicals (•OH). The substrate 3,3′,5,5′-tetramethylbenzidine (TMB) was concomitantly oxidized to blue product ox-TMB by •OH. This colorimetric sensor easily differentiates *S. aureus* from *E. coli* and *B. subtilis* within 30 min, with a LOD of 4 × 10^2^ CFU/mL. Indeed, nanoenzymes are widely used in analytical chemistry (Wang et al., 2018; Huang et al., 2019), and the development of biosensors for the bacterial detection based on Au NC with mimic enzyme-like catalytic activities has very attractive application prospects.

### Sensor Arrays

**Figure 5:**
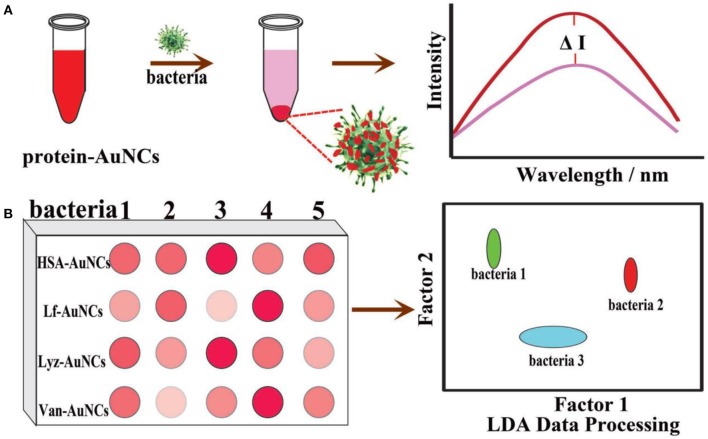
Schematic illustration of protein-Au NC-based fluorescence sensor array for discrimination of various bacteria. **(A)** The fluorescence intensity of protein-Au NCs was significantly reduced in the presence of bacteria. **(B)** A schematic fluorescence pattern generated from the different responses of the protein-Au NCs probes toward bacteria. Reproduced from Ji et al. (2018) with permission from Wiley-VCH Verlag & Co. KGaA, Weinheim.

To achieve simultaneous detection of multiple bacteria, sensor arrays based on Au NCs have also been developed. For instance, Qu and coworkers designed and prepared a bacterial sensor array based on the integration of HSA-Au NCs, lysozyme (Lyz)-Au NCs, lactoferrin (Lf)-Au NCs, and vancomycin decorated HSA-Au NCs (Van-Au NCs) (Figure 5) (Ji et al., 2018). HSA-Au NCs are selected based on the interaction between the peptide motifs on the surface of HSA and the bacterial cell wall (Chan and Chen, 2012). Lysozyme can recognize and kill bacteria by binding to the cell surface polysaccharide (Vocadlo et al., 2001). Lf-Au NCs can serve as a probe since many bacteria can express lactoferrin receptors with high affinity to lactoferrin (Xavier et al., 2010). The strong affinity of Van to D-alanyl-D-alanine dipeptide on bacterial cell walls endows Van-Au NCs with a high binding affinity to both gram-positive and gram-negative bacteria (Xing et al., 2002; Chung et al., 2011). The subtle changes in the physicochemical properties on different bacterial surfaces would induce different interactions with the probes in the sensor array. Based on the sensor array, six types of bacteria, including *Alcaligenes faecalis, B. subtilis, S. aureus*, MRSA, *E. coli*, and kanamycin-resistant *E. coli* were distinguished on the sensor array. Similarly, a bacterial sensor array based on metal ion modified Au NCs was established (Wu et al., 2018). In another study, Yang and coworkers fabricated a sensor array based on Ag-Au alloy NC-Au NP composite for the discrimination of sulfur-oxidizing bacteria (Yang et al., 2019). The non-sulfur (*S. aureus* and *E. coli*) and sulfur-oxidizing bacteria (*Citreicella thiooxidans, Thiobacimonas profunda*, and *Acidithiobacillus caldus*) were well distinguished at a level of OD_600_ = 0.005. In summary, the development of these sensor arrays might offer new perspectives for analyzing intricate bacterial infections.

As mentioned above, Au NCs-based bacterial biosensors may provide a promising alternative platform for detection and discrimination of pathogenic bacteria. However, there are still great challenges that limited their practical applications. First of all, the stability of Au NCs is essential in their practical use. In general, the as-synthesized Au NCs are not comprised of a single component, and they are usually a complicated mixture system of multiple Au species, leading to the bacterial biosensors based on Au NCs are less reproducible. To overcome this problem, atomic precision controlled synthesis of Au NCs could be utilized to improve their reproducibility. Secondly, the detection selectivity should be further improved to work in the biomatrices. Functionalization of fluorescent Au NCs with specific recognition motifs via surface chemistry may be helpful to address this problem. In addition, fundamental understanding of the fluorescence mechanisms of Au NCs is vital to develop bacterial sensors. In this aspect, understanding of their photoluminescence mechanisms such as FRET will help to optimize the detection strategy (Yu et al., 2017).

## Au NCs as Antibacterial Agents

In addition to bacterial detection, ultrasmall Au NCs are also developed as an innovative nanomedicine for the treatment of multidrug-resistant bacterial infections in recent years. The antibacterial activity of these Au NCs usually results from the antibiotics delivery, generation of reactive oxygen species (ROS), and damage of cell membrane and cellular contents. Usually the antibacterial activity is affected by the size and surface chemistry of NCs. In this section, we will summarize in details the progress made by Au NCs in the treatment of bacterial infections and classify them into different antibacterial systems based on the fundamental components of Au NCs, including antibiotic-Au NCs systems, antimicrobial peptide-Au NCs systems, small molecule-Au NCs systems, macromolecule-Au NCs systems, and Au NCs-containing combination systems (Table 1).

**Table 1:** Antibacterial applications of Au NCs.

**Formulations**	**Target pathogen**	**Antibacterial mechanism**	**References**
**ANTIBIOTIC-Au NCs SYSTEMS**			
Cefradine-labeled Au_8_ clusters	*E. coli*	Increased cefradine bioavailability	Khandelwal et al., 2015
Vancomycin-loaded Pep-Au NCs	*S. aureus*	Increased antibacterial activity by drug encapsulation; spontaneous released vancomycin	Li et al., 2018
Bacitracin templated Au NCs	*S. aureus*	ROS production; prolonged bacitracin release	Wang S. et al., 2019
Lys-Au NCs-Amp	MRSA and its persister	Increased concentration of ampicillin at the action site; the multivalent presentation and the enhanced permeation of ampicillin via lysozyme-induced cell lysis	Kalita et al., 2018
Vancomycin templated Au NCs	*E. coli*; *S. aureus*	Increased vancomycin bioavailability	Liang et al., 2018
Chloramphenicol loaded Au NCs	*E. coli*	Prolonged drug release	Liu P. et al., 2015
**ANTIMICROBIAL PEPTIDE-Au NCs SYSTEMS**			
SFT/DT-Au NDs	Gram-negative bacteria; Gram-positive bacteria	Synergistic effect; bacterial membrane disruption	Chen W. Y. et al., 2015
Cysteine-terminated antimicrobial peptide templated Au NCs	Gram-negative bacteria; Gram-positive bacteria	pH-responsive charge reversal; disruption of the bacterial membrane	Pranantyo et al., 2019
Dap-Au NCs	MRSA	Synergistic effect; bacterial membrane disruption; ROS production; DNA damage	Zheng Y. et al., 2019
**SMALL MOLECULE-Au NCs SYSTEMS**			
Cys-Au NCs	*E. coli*	ROS production	Chang et al., 2019
Au_25_(MHA)_18_	Gram-negative bacteria; Gram-positive bacteria	Bacterial membrane disruption; ROS production; induced metabolic imbalance	Zheng K. et al., 2017
thiolated Au NCs	*S. aureus*	ROS production; bacterial membrane disruption	Zheng et al., 2018a
AuDAMP	Gram-negative bacteria; Gram-positive bacteria	Bacterial membrane disruption; ROS production; DNA damage	Zheng et al., 2018c
Man-Au NDs	*E. coli*	Agglutination	Tseng et al., 2011
AuMS	Gram-negative bacteria; Gram-positive bacteria	Bacterial membrane disruption; biofilm inhibition	Boda et al., 2015
QA-Au NCs	MRSA	ROS production; bacterial membrane disruption; ATP metabolic disturbance	Xie et al., 2018
**MACROMOLECULE-Au NCs SYSTEMS**			
antiSAIgG-BSA- PS- Au NCs	*S. aureus*	Photodynamic inactivation	Khlebtsov et al., 2015
lysozyme-Au NCs	*A. baumannii*; *E. faecalis*	Increased bioavailability	Chen et al., 2010
DPAu/AMD	*E. coli*; *S. aureus*	Increased bioavailability; prolonged drug release	Setyawati et al., 2014
dendrimer-Au NCs	Gram-negative bacteria	Retard endotoxin activity	Liao et al., 2018
**Au NCs-CONTAINING COMBINATION SYSTEMS**			
TiO2/graphene/Au NC nanocomposites	*E. coli*; *S. aureus*	Enhanced ROS production	Zhou et al., 2019
Au NCs/Ho-GO nanosheets	Gram-negative bacteria; Gram-positive bacteria	Synergistic effect; bacterial membrane disruption; ROS production; induced metabolic imbalance; physical piercing	Zheng K. et al., 2019
Au NCs/CS	Gram-negative bacteria; Gram-positive bacteria	Bacterial membrane disruption	Girija et al., 2019
Kanamycin-loaded MSN-Au NC@Lys	*E. coli*	Increased bioavailability; prolonged drug release	Alsaiari et al., 2017
Prot/MTU-Au NCs	*E. coli*; *S. aureus*	Synergistic effect; enhanced ROS production	Zhu et al., 2019

### Antibiotic-Au NCs Systems

The abuse of antibiotics and low utilization rate are one of the main causes of antibiotic resistance (Li X. et al., 2019). One feasible solution for reducing the abuse of antibiotics and improving the effect involves the use of assembled structures that have adjustable antibacterial activity. As a versatile platform, Au NCs can be widely used in antibiotic loading to improve internalization of antibiotics into bacteria, thereby improving the efficacy of antibiotics. In addition, some Au NCs possess inherent antibacterial activity, and may exert certain polyvalent and synergistic effects through antibiotic loading to enhance the antibacterial activity of nanosystems (Zhang et al., 2014; Zheng Y. et al., 2019). For example, Kalita et al. developed a potent antibacterial hybrid prepared through surface functionalization of lysozyme-capped Au NCs (Lys-Au NCs) with β-lactam antibiotic ampicillin (Lys-Au NCs-Amp) (Kalita et al., 2018). The antibacterial hybrid not only reverses the MRSA resistance toward ampicillin but also exhibits enhanced antimicrobial activity against non-resistant bacterial strains. With the help of cis-2-decenoic acid, Lys-Au NCs-Amp can also inhibit the MRSA persister, a dormant body of bacteria. This antibacterial hybrid may eradicate MRSA infections from difficult-to-treat diabetic wound of rat and accelerate the healing process. Antibacterial mechanism studies have shown that the antibiotic effect of Lys-Au NCs-Amp against MRSA and its persister is due to the increased concentration of ampicillin at the action site, the multivalent presentation and the enhanced permeation of ampicillin via lysozyme-mediated cell lysis. In another study, the self-regulated vancomycin loading and release capabilities of custom-designed pentapeptide-capped Au NCs (Pep-Au NCs) were developed on basis of the strong binding affinity of vancomycin with D-alanine-D-alanine termini (Li et al., 2018). The self-assembly Au NCs super-structure can spontaneously release vancomycin upon exposure to gram-positive bacteria due to the stronger binding affinity of vancomycin with bacteria than that with Pep-Au NCs. Note that the formation of this structure does not mitigate the efficacy of the vancomycin. The on-demand drug release of Pep-Au NCs avoids the systemic distribution of vancomycin and reduces the potential side effect. Besides as a cargo, vancomycin can also be directly used as a reducing agent and template to fabricate the water-soluble, monodispersed Au NC (Liang et al., 2018), which have excellent antibacterial activities toward both gram-positive and gram-negative bacteria. This encouraging result suggests that loading antibiotics with Au NCs may broaden the antibacterial spectrum of antibiotics themselves, rendering their broader antibacterial applications. In addition, bacitracin-templated Au NCs (Wang S. et al., 2019), cefradine-synthesized Au NCs (Khandelwal et al., 2015), and chloramphenicol-loaded Au NCs (Liu P. et al., 2015) have also been developed for the treatment of multidrug resistant infections.

### Antimicrobial Peptide-Au NCs Systems

**Figure 6:**
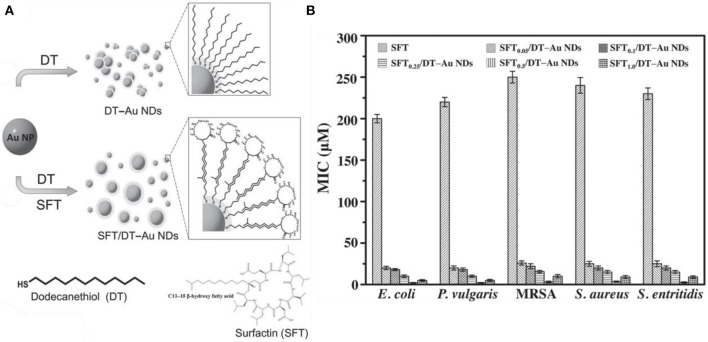
**(A)** Synthesis of photoluminescent SFT/DT-Au NDs. **(B)** Comparison of MICs (in terms of the concentration of SFT) of SFT, SFT_0.05_/DT-Au NDs, SFT_0.1_/DT-Au NDs, SFT_0.25_/DT-Au NDs, SFT_0.5_/DT-Au NDs, and SFT_1.0_/DT-Au NDs against *E. coli, P. vulgaris*, MRSA, *S. aureus*, and *Salmonella enterica*, respectively. Error bars represent the standard deviation of three repeated measurements. Reproduced from citetbib9 with permission from Wiley-VCH Verlag & Co. KGaA, Weinheim.

**Figure 7:**
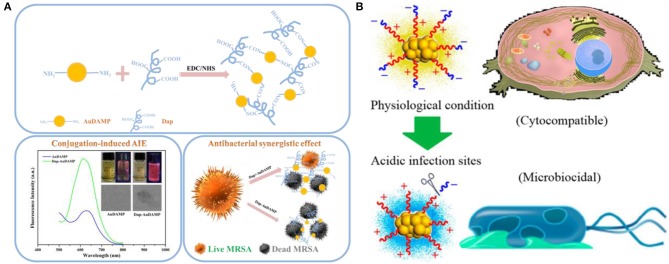
**(A)** Schematic illustrations of the conjugation strategy for antibacterial Au NCs and Dap, conjugation-induced aggregation-induced emission enhancement, and antibacterial synergistic effect. Reproduced from Zheng Y. et al. (2019) with permission from Elsevier Inc. **(B)** Schematic illustration of antimicrobial peptide-reduced Au NCs with charge-reversal moieties for antibacterial application. Reproduced from Pranantyo et al. (2019) with permission from American Chemical Society.

Except antibiotics, antimicrobial peptide-functionalized Au NCs have also been developed as promising therapeutic for multidrug-resistant infections. Antimicrobial peptides are produced by organisms to defend themselves against pathogenic bacteria (Rajchakit and Sarojini, 2017). The common and generally accepted mechanism of action of antimicrobial peptides is perturbation or complete lysis of bacterial membranes relying on their distinctive amino sequences that can insert into bacterial membrane (Hancock and Sahl, 2006; Hassan et al., 2012; Hilchie et al., 2013). In a previous study, Chen et al. prepared a ~2.5 nm Au NCs-based antibacterial structure via etching and co-deposition of 1-dodecanethiol (DT) and antimicrobial peptide surfaction (SFT) on gold NPs (Figure 6A) (Chen W. Y. et al., 2015). The as-synthesized SFT/DT-Au nanodots (NDs, ~2.5 nm) show significant antibacterial behavior and their antibacterial activities are highly dependent on the density of SFT on NDs. Relative to SFT alone, the antibacterial hybrid exhibit stronger antibacterial activity to multidrug-resistant bacteria (Figure 6B). The total antibacterial activity is mainly attributed to the synergistic effect of SFT and NDs on the disruption of the bacterial cell membrane. In our recent study, we developed an effective antibacterial hybrid (Dap-Au NCs) by incorporating antimicrobial peptide (daptomycin, Dap) and antibacterial Au NCs (Zheng et al., 2018c; Zheng Y. et al., 2019). The antibacterial hybrid could high-efficiently damage bacterial cell membrane because Dap moiety may induce the creation of holes on the cell membranes, and motivate the entry of Dap-Au NCs inside bacteria and even lead to serious DNA destruction (Figure 7A). In addition, Dap-Au NCs can also promote the generation of free radicals such as ROS within bacteria, which may also limit the evolution of drug resistance in bacteria. In another work, cysteine-terminated antimicrobial peptide was employed as a reducing ligand to prepare Au NCs (Pranantyo et al., 2019). The citraconyl amide on the surface of Au NCs could auto-cleave to re-expose the cationic amine at low pH. As a result, the NCs are stable and non-cytotoxic under physiological conditions, but can switch into a cationic bactericidal mode in an acidic environment that is commonly encountered at bacterial infection areas (Figure 7B).

### Small Molecule-Au NCs Systems

**Figure 8:**
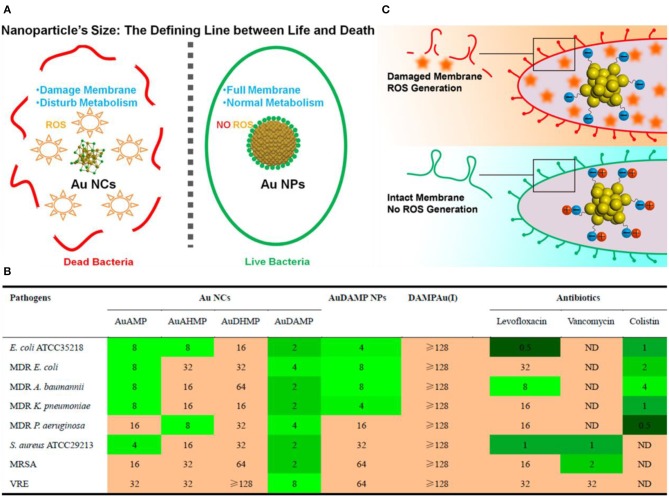
**(A)** Schematic illustration of the size regulation of Au NPs to significantly affect their antibacterial properties. Reproduced from Zheng K. et al. (2017) with permission from American Chemical Society. **(B)** Antibacterial activities of mercaptopyrimidine-conjugated Au NCs indicated with MIC (μg/mL). Here DAMPAu(I) is the precursor complex during the synthesis of Au NCs. Reproduced from Zheng et al. (2018c) with permission from American Chemical Society. **(C)** Surface ligand chemistry of gold nanoclusters determines their antimicrobial ability. Reproduced from Zheng et al. (2018a) with permission from American Chemical Society.

**Figure 9:**
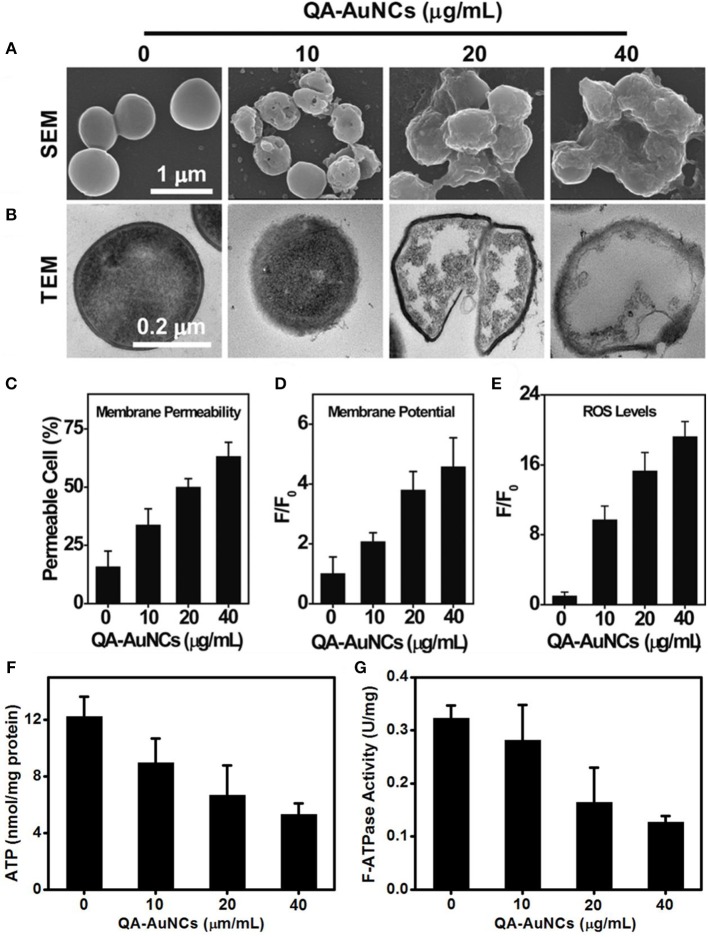
QA-Au NCs combat bacteria through a multipath mechanism. **(A)** Scanning electron microscopy (SEM) and **(B)** TEM images showing the morphological changes of *S. aureus* after treatment with QA-Au NCs. The administration of QA-Au NCs leads to an increase in the membrane permeability **(C)**, a dissipation of the membrane potential **(D)** and the generation of ROS **(E)**. The intracellular ATP level **(F)** and F-type ATPase activity **(G)** of *S. aureus* decrease upon treatment with increasing concentrations of QA-Au NCs. Reproduced from Xie et al. (2018) with permission from Wiley-VCH Verlag & Co. KGaA, Weinheim.

Compared with passive drug carriers, non-antibiotic small molecule-functionalized Au NCs can directly obtain antibacterial ability through precise size and surface chemistry regulations, which showed great potential as an alternative for commercial antibiotics. In a pioneering work, Zheng et al. demonstrated that ultasmall Au NCs (<2 nm) may own antibacterial activity that not observed for large-sized Au NPs with same ligands (Zheng K. et al., 2017). The synthesized atomic precision 6-mercaptohexanoic acid (MHA)-templated Au NCs (Au_25_MHA_18_) showed a wide-spectrum antibacterial activity and exhibited interactions with both gram-negative and gram-positive bacteria to induce intracellular metabolic disorders after the internalization of Au_25_MHA_18_, and result in an increase of intracellular ROS generation that killed bacteria consequently (Figure 8A). However, the large-sized MHA-Au NPs (~6 nm) cannot induce ROS generation and therefore did not possess antibacterial capability. Indeed, the induction of ROS generation is the dominant antibacterial mechanism of action of Au NCs (Chang et al., 2019). Similar circumstances have been witnessed in our recent work. We demonstrated that mercaptopyrimidine analogs templated Au NCs can serve as potent nanoantibiotics for ESKAPE superbugs (Zheng et al., 2018c). Mercaptopyrimidine analogs, including 4,6-dihydroxyl-2-mercaptopyrimidine (DHMP), 4-amino-6-hydroxyl-2-mercaptopyrimidine (AHMP), 4,6-diamino-2-mercaptopyrimidine (DAMP), and 4-amino-2-mercaptopyrimidine (AMP), were employed as templates and reducing agents to prepare Au NCs. Unlike large-sized Au NPs, the as-prepared Au NCs especially AuDAMP possess excellent antibacterial capabilities against both gram-negative and gram-positive bacteria (Figure 8B). The Au NCs kill ESKAPE via a combined mechanism including cell membrane damage, DNA destruction, and ROS production. Moreover, the induction of ROS generation in bacteria is mainly attributed to intrinsic oxidase- and peroxidase-like catalysis by Au NCs. In contrast, large-sized AuDAMP NPs exhibit relatively weak antibacterial activity due to their weak enzyme mimic activity. Broadening the antibacterial spectrum of Au NCs by co-functionalizing albumin and DAMP has also been reported (Sun et al., 2019). Quaternary ammonium-functionalized Au NCs (QA-Au NCs) have been utilized to treat bacterial infections through the combined physicochemical mechanisms including cell membrane disruption, ROS generation, and disturbance of intracellular metabolic pathways (Figure 9) (Xie et al., 2018). These NCs can specifically target and kill antibiotic-resistant gram-positive superbugs including MRSA and vancomycin-resistant *Enterococcus*. Mannose-protected Au NCs were also found to selectively and efficiently inhibit the proliferation of *E. coli* through Au NCs-induced agglutination (Tseng et al., 2011). Furthermore, the use of antibacterial Au NCs for biofilm inhibition of multidrug-resistant bacteria has also been demonstrated (Boda et al., 2015).

In addition to size effects, the surface ligand chemistry of Au NCs also profoundly affects their antibacterial properties. In general, antibacterial agents with positive surface charges are considered to lead higher antibacterial abilities (El Badawy et al., 2011; Chen W. Y. et al., 2015; Le Ouay and Stellacci, 2015; Zheng et al., 2018c). However, Zheng et al. obtained the opposite results when using Au_25_(SR)_18_ NCs (SR means thiolate ligands) (Zheng et al., 2018a). The molecular features and the surface properties of Au_25_(SR)_18_ NCs could be precisely tailored at the atomic level, producing a series of Au NCs with same Au atom numbers but different surface properties. By adjusting the type and ratio of surface ligands on Au_25_(SR)_18_, more negatively charged Au_25_(SR)_18_ would produce more ROS, leading to a better bacterial killing efficiency (Figure 8C). This unexpected result indicates the intricacies of the nano-bio interactions and may offer some inspiration on the design of high-performance Au NCs-based antibacterial drugs.

### Macromolecule-Au NCs Systems

**Figure 10:**
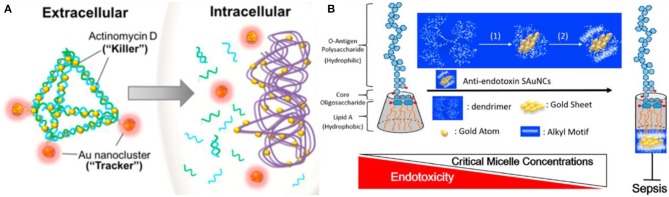
**(A)** Schematic illustration of DPAu/AMD as an image-guided nanotheranostic agent. Reproduced from Setyawati et al. (2014) with permission from American Chemical Society. **(B)** A simple model representing the possible correlation between the packing density of lipid A of lipopolysaccharide and sepsis progression in the presence of Au NCs. Reproduced from Liao et al. (2018) with permission from American Chemical Society.

Macromolecules such as proteins, DNA, and dendrimers are also commonly used as the surface ligands attached to Au NCs for antibacterial treatments. Functionalization of macromolecules endowed potent antibacterial therapeutic capabilities on Au NCs. Chen et al. have prepared lysozyme-directed Au NCs as potential antibacterial agent for multidrug-resistant bacteria, including notorious pandrug resistant *A. baumannii* (Chen et al., 2010). Setyawati et al. have used DNA nanopyramid as the scaffold to intercalate red-emissive glutathione-capped Au NCs and actinomycin D to form an image-guided nanoantibiotics (DPAu/AMD) (Figure 10A) (Setyawati et al., 2014). The nanotheranostic agents of DPAu/AMD show a significant antibacterial efficiency and have been applied for the simultaneous diagnosis and treatment of *E. coli* and *S. aureus* infections. Furthermore, Liao and coworkers have constructed dendrimer-capped Au NCs that can effectively retard endotoxin activity to protect against sepsis (Liao et al., 2018). The retardant consists of an Au NC that acts as a flake-like substrate and a coating of short alkyl motifs that serve as an adhesive to dock with lipopolysaccharide by compacting the intramolecular hydrocarbon chain-chain distance of lipid A, which is an endotoxicity active site that can cause overwhelming cytokine induction resulting in sepsis progression (Figure 10B). The treatment of the antiendotoxin Au NCs prominently extended the survival time in lipopolysaccharide-induced septicemic mouse. This work might present a potential treatment for the early prophylaxis of septicemia. In addition, a photodynamic antibacterial treatment strategy based on Au NCs has also been developed through photosensitizer conjugated BSA-capped Au NCs (Khlebtsov et al., 2015).

### Au NCs-Containing Combination Systems

**Figure 11:**
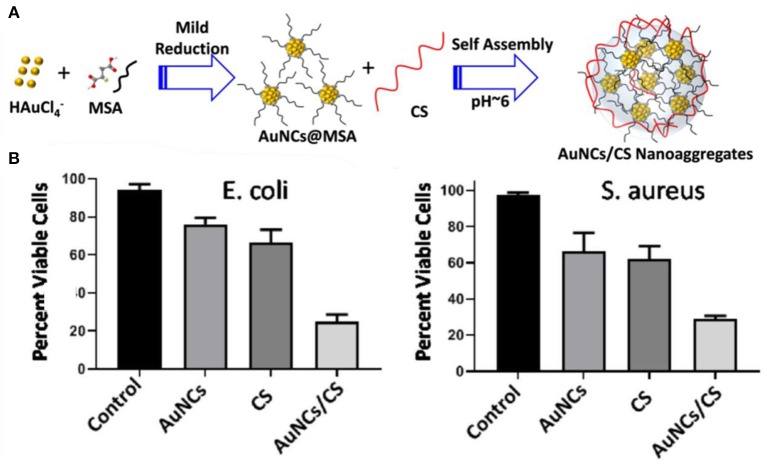
**(A)** Schematic representation of the formation of AuNCs/CS nanoaggregates. **(B)** The viability of *E. coli* and *S. aureus* post treatment with nanoaggregates. Reproduced from Girija et al. (2019) with permission from Wiley-VCH Verlag & Co. KGaA, Weinheim.

In order to improve the antibacterial performances, antibacterial composites based on Au NCs have also been developed as nanoantibiotics. For instance, Zheng et al. established a synergistic antibacterial agent through assembly of paramagnetic Ho ions and Au NCs onto graphene oxide (GO) nanosheets (Zheng K. et al., 2019). GO is a new type of antibiotic substance combined with multiple mechanisms, and its rich functional groups enable the functionalization of nanomaterials to further ameliorate antimicrobial performances (Ji et al., 2016; Xia et al., 2019). The assembled nanostructures could be effectively piercing the bacteria. Meanwhile, the decorated Au NCs could efficiently induce bacteria to generate high-concentration ROS, severely interfere with bacterial metabolism, leading to the death of multidrug-resistant bacteria. TiO_2_/graphene/Au NC nanocomposites were also developed to ameliorate the antimicrobial capability of Au NCs under sunlight (Zhou et al., 2019). Conjugation of graphene and Au NCs into TiO_2_ NPs can dramatically improve the solar energy utilization efficiency and increase ROS levels, resulting in enhanced antibacterial activity. Alsaiari et al. designed and fabricated an intelligent antimicrobial mixed-matrix membrane coating comprising lysozyme-Au NCs and kanamycin as nanofillers (Alsaiari et al., 2017). The mixed-matrix coating can successfully treat healthcare-associated infections. In another study, protamine (Prot) functionalized Au NCs (Prot-Au NCs) with a highly stable ability to load positively charged antibacterial agents were developed, which may penetrate into the bacteria, thereby enhancing the ability to treat bacterial infections (Zhu et al., 2019). In addition, chitosan (CS)-induced antibacterial Au NCs nanoaggregates have also been found to significantly enhance antibacterial activity and facilitate rapid wound healing compared with their individual components (Figure 11) (Girija et al., 2019). Indeed, it appears that aggregation of nanomaterials can significantly improve their physicochemical properties and subsequently affect their therapeutic effects (Goswami et al., 2016; Qin et al., 2019). These investigations provide new options for improving the antibacterial properties of Au NCs.

As a kind of innovative antibacterial nanomedicine, Au NCs have very attractive prospects in dealing with increasingly severe multidrug resistant infections. However, considering that the research of Au NCs as an antibacterial agent is in its infancy, there still remain several problems to be solved. Firstly, the synthesis of Au NCs with antibacterial activity at atomic precision is still a major challenge. Although the current research found that reducing the size of Au nanomaterials can make them have antibacterial capabilities, the structure of Au NCs is still not unique, which is a major obstacle to understanding the accurate antibacterial mechanism of Au NCs. Therefore, it is imperative to synthesize water-soluble Au NCs with a confirmed structure for the evaluation of antibacterial activity. Second, the general antibacterial mechanisms of Au NCs need further investigation. Although various mechanisms are proposed to explain the antibacterial property of Au NCs up to now, the metabolisms of Au NCs in bacterial cells are still needed for in-depth understanding their antibacterial activity by both experimental and theoretical studies. In addition, the possible development of bacterial resistance to Au NCs needs to be concerned. Although no reports of bacterial resistance to Au NCs have been reported, bacteria resistant to antibacterial Ag NPs have emerged (Panácek et al., 2018). Therefore, it is necessary to study the development of bacteria resistance to antibacterial Au NCs in a longer period by using genome-wide analysis. Finally, the biological safety of antibacterial Au NCs on animals, especially the effect on intestinal flora needs further exploration (Li J. et al., 2019; Wang L. et al., 2019).

## Conclusions

In summary, we have attempted to present a review of the recent efforts on Au NCs from the multipath bacterial diagnostics and treatment. Due to the unique physicochemical properties, excellent biocompatibility, as well as advantages of easy surface functionalization of Au NCs, the recent mushrooming in fabrication and modification of Au NCs has empowered the exploitation of these nanomaterials for applications in selective detection of bacteria and infection treatment. In terms of bacterial detection, label-free detection strategies, specific molecular recognition strategies, and sensor arrays based on gold nanoclusters, have been established. In terms of bacterial infection treatment, Au NCs-based different antibacterial systems, including antibiotic-Au NCs systems, antimicrobial peptide-Au NCs systems, small molecule-Au NCs systems, macromolecule-Au NCs systems, and Au NCs-containing combination systems have been used for the treatment of multidrug-resistant bacterial infections. These studies reveal that ultrasmall Au NCs can offer promising opportunity in biomedicine to promote the mushrooming in this field. With the continuing development to unravel the structure-function relationships, we believe that the ultrasmall Au NCs will eventually serve as an important platform for bacterial detection and infection treatment.

## Author Contributions

MT and YZ performed literature search as well as the majority of the authoring and editing. JZ and CY performed the literature arrangement and writing for future perspectives. HJ proposed topic of paper and provided overall direction of manuscript.

### Conflict of Interest

The authors declare that the research was conducted in the absence of any commercial or financial relationships that could be construed as a potential conflict of interest.
